# Self-Assembled Cellulose Nanocrystal–MXene Hybrid Film for Acceleration Sensing

**DOI:** 10.1021/acs.nanolett.5c02824

**Published:** 2025-10-29

**Authors:** Omer Shoseyov, Daniel Voignac, Shylee Belsey, Danielle Sviri, Shira Yochelis, Maxim Sokol, Oded Shoseyov, Yossi Paltiel

**Affiliations:** † Institute of Applied Physics, 26742 The Hebrew University of Jerusalem, Jerusalem 9190401, Israel; ‡ The Robert H. Smith Institute of Plant Sciences and Genetics in Agriculture, Robert H. Smith Faculty of Agriculture, Food and Environment, The Hebrew University of Jerusalem, Rehovot 7612001, Israel; § The Center for Nanoscience and Nanotechnology, The Hebrew University of Jerusalem, Jerusalem 9190401, Israel; ∥ Department of Materials Science and Engineering, 26745 Tel Aviv University, Ramat Aviv 6997801, Israel

**Keywords:** Self-Assembled Acceleration Sensor, Spreadable Accelerometer, Flexible Piezo Accelerometer, Biodegradable Sensor

## Abstract

Advances in robotics and micromechanical systems demand miniaturized, low-cost electromechanical sensors. Conventional micro-electromechanical systems (MEMS) rely on complex, expensive top-down fabrication, limiting scalability. Here, we introduce a bottom-up approach for fabricating a flexible acceleration sensor using cellulose nanocrystal (CNC) films combined with conductive 2D MXene nanosheets. The self-assembled hybrid film exhibits sensitivity to acceleration, enabling precise three-axis motion detection. Functioning like a flexible field-effect transistor, the device uses acceleration-induced film deformation to generate charge separation in the chiral piezo films, producing a gating effect with measurable voltage shifts proportional to applied acceleration. This piezoelectric response allows real-time accurate motion tracking. Unlike conventional sensors, the device exhibits nonlinear behavior and is insensitive to the motion direction. Our approach offers a cost-effective solution for applications requiring dynamic motion detection and precise acceleration quantification, while simplifying fabrication and expanding the possibilities for next-generation nano and micro sensing technologies.

## Graphical abstract

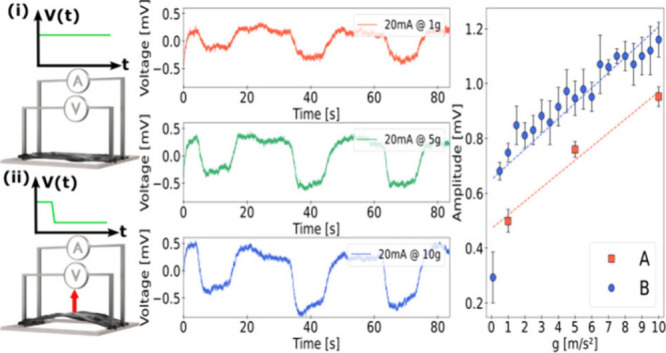

Acceleration sensors play a critical role in modern technology, enabling precise motion detection in applications ranging from consumer electronics and automotive safety to aerospace engineering and structural health monitoring.^1–4^ These sensors are essential for tracking motion, detecting vibrations, and providing feedback in systems that require stability, control, or real-time response. The growing demand for high-performance acceleration sensors has led to significant advancements in sensor technologies, including miniaturized, high-sensitivity devices capable of operating in extreme environments.^5^ Among the most widely used acceleration sensors are micro-electromechanical system (MEMS)-based devices,^6^ which rely on capacitive,^7^ piezoresistive,^8^ or resonant detection mechanisms.^9^ MEMS accelerometers are favored for their small size, low power consumption, and compatibility with integrated circuits, making them ideal for applications in smartphones, wearable devices, and automotive airbag systems.^10^ However, despite their advantages, MEMS accelerometers suffer from several limitations, including limited sensitivity at low frequencies,^11^ temperature-dependent performance,^12^ and mechanical drift over time.^13^ Furthermore, their fabrication process requires advanced lithographic techniques,^14^ increasing production costs and limiting design flexibility. Piezoelectric acceleration sensors provide an alternative approach to motion sensing by leveraging the intrinsic ability of certain materials to generate an electric charge in response to mechanical stress.^15^ These sensors are widely used in high-frequency vibration analysis,^16^ impact detection, and aerospace applications due to their high dynamic range, fast response time, and robustness in extreme conditions.^5^ Traditional piezoelectric accelerometers are often based on ceramic materials such as lead zirconate titanate (PZT),^17^ which exhibit strong piezoelectric effects but suffer from brittleness and environmental concerns related to lead content.^18^ As an alternative, flexible piezoelectric polymers such as polyvinylidene fluoride (PVDF) offer improved mechanical robustness, enabling their use in dynamic or deformable environments, and can be readily integrated into nonrigid substrates, wearable devices, or curved structural surfaces.^19^ However, conventional piezoelectric sensors typically require charge amplifiers and specialized signal processing, adding complexity to their implementation.^20^

In the present study, a self-assembled cellulose nanocrystals (CNCs) and MXene hybrid film (CMHF) is used for acceleration sensing. Cellulose, Earth’s most abundant biopolymer, has long been utilized in the electronics industry due to its excellent dielectric properties.^21^ In recent years, there has been an increasing interest in cellulose-based nanoparticles. At the nanoscale, cellulose exists in both crystalline and amorphous forms, with CNCs being derived through a straightforward sulfuric acid hydrolysis process.^22^ These nanocrystals typically take the form of rod-like structures, approximately 200 nm in length and 5 nm in diameter.^23^ CNCs are primarily sourced from plants and plant-derived materials, but they can also be biosynthesized by certain bacteria and tunicates.^22^ Their exceptional properties make them an attractive material for applications spanning from commercial packaging^24^ to advanced electronics.^25^ Recognized as a sustainable alternative to synthetic polymers, CNCs are biodegradable, renewable, and recyclable. Due to their water dispersibility, CNCs enable facile and environmentally friendly processing.^26,27^ Upon water evaporation, they self-assemble into strong, flexible, and transparent films, exhibiting a chiral nematic arrangement at the nano- and microscale.^28^ The sulfuric acid hydrolysis process imparts a negative surface charge to the CNCs, allowing their use in Pickering emulsions and other dispersion mechanisms, which facilitate the dispersion of otherwise incompatible particles and molecules in aqueous systems.^29,30^ In addition to their dielectric properties reported with a relative permittivity ranging from 3 to 8 CNCs also exhibit piezoelectric behavior.^31,32^ Furthermore, the highly organized structure of self-assembled CNC films provides a unique templating effect, enabling a controlled spatial arrangement of nanoparticles. This organization allows for precise nanoscale interactions, facilitating applications in quantum-based technologies and electronics.^33^

MXenes are a chemically versatile class of two-dimensional (2D)^34^ materials that can be readily scaled up and integrated with CNCs. They have a general formula of M_*n*+1_X_*n*_T_*z*_, where *n* ranges from 1 to 4, and are derived from their three-dimensional (3D) bulk layered counterparts known as MAX phases (M_*n*+1_AX_*n*_). In this structure, M represents an early transition metal such as titanium, niobium, or vanadium, A is a group 13 or 14 element, such as aluminum, silicon, or gallium, which is selectively removed through etching, X is either carbon and/or nitrogen, and T_*z*_ denotes surface terminations such as −O, −F, or −OH. Compared to other 2D materials, MXenes offer several advantages, including relatively low cost, ease of scalable synthesis, and stable dispersion in water.^35^ These properties make MXenes highly attractive for various applications, particularly in combination with CNC-based materials.^36^

In this work, we utilized hybrid self-assembled MXene CNC smearable sheets to realize acceleration sensors. The sensor is subjected to various radial acceleration profiles, enabling the evaluation of its response to dynamic motion (see Figure 2b).

**Figure 1:**
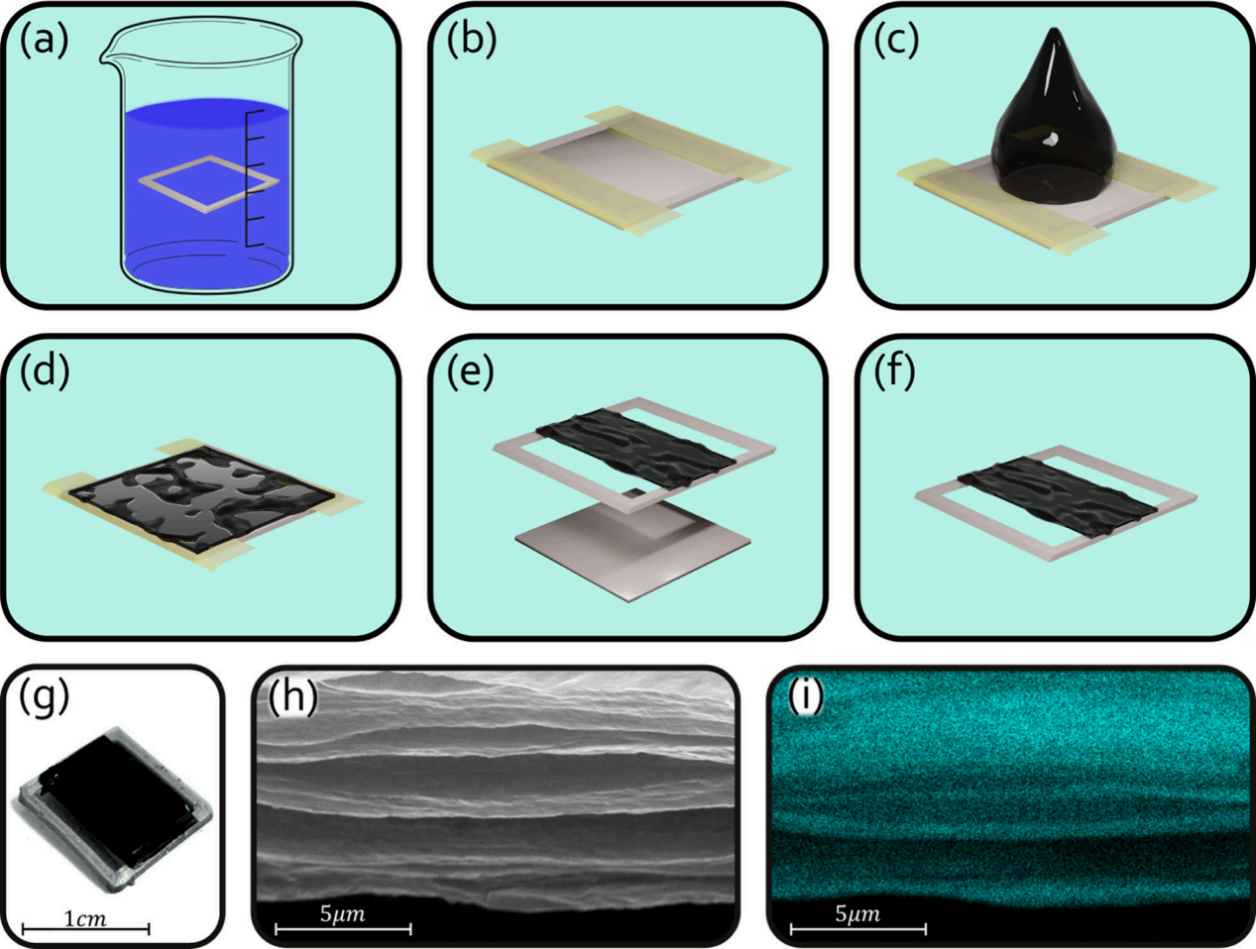
CNC–MXene hybrid film fabrication process. (a) A polylactic acid (PLA) 10 × 10 × 1 mm frame is immersed in 1 wt % polyethylenimine solution, (b) an inner substrate is added to the frame and taped using Kapton, (c) a CNC–MXene dispersion is drop casted on the exposed areas, (d) the sample is left to dry, (e) the inner substrate is removed, and (f) a suspended CNC–MXene hybrid film results. (g) Optical image of a CNC–MXene hybrid film device. (h) Scanning electron microscope (SEM) image of the film. (i) EDX image of the film showing the Ti dispersion in the film.

**Figure 2:**
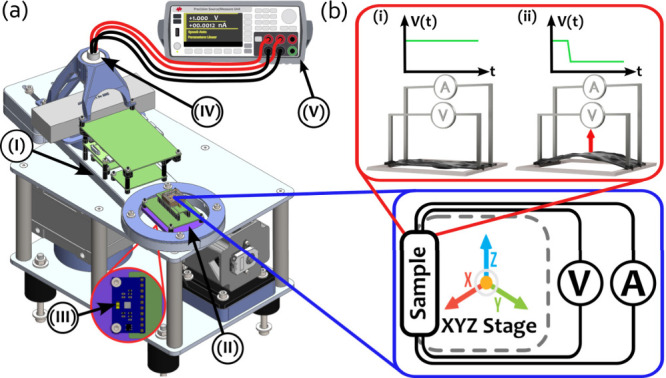
Device measurement setup. (a) Custom rotational measurement system. (I) Rotating arm, (II) XYZ stage, (III) MEMS reference accelerometer, (IV) slip ring, (V) source meter unit (SMU). (b, top) Illustration of the active sensing component in the CNC–MXene hybrid film sensor. (i) The CNC–MXene hybrid film is suspended on a PLA frame and connected to a source-measure unit in a four-probe configuration. (ii) Application of external force (indicated by the red arrow) causes the film to bend, inducing charge separation that acts as a gate to the conducting MXene sheet, changing the measured voltage. (b, bottom) XYZ stage allows orientation of the device in various orientations with respect to the rotation plane.

Fabrication of the CMHF device includes the following steps, a polylactic acid (PLA, Spider 3D, Israel) frame and casting holder were printed on a fuse deposition modeling 3D printer (Creality Ender 3 v2, China). The frame is a 10 × 10 × 1 mm hollow frame with 1 mm edges, and the inner surface is 7.8 × 7.8 × 1 mm. The outer frame was immersed for 10 min in a 1 wt % polyethylenimine (PEI, Sigma, Israel) solution to facilitate CNC adhesion (Figure 1a). Frames were then rinsed in deionized water (DW) to leave only a PEI monolayer on the surface. The inner substrates were taped with a polyamide tape (Kapton) to ensure that the CNC does not adhere after drying. After inserting the core substrate in the frame, two strips of 1 mm tape were placed on opposing edges and used as masks (Figure 1b). A 20 μL amount of CNC–MXene dispersion was drop cast to cover the exposed area across the central substrate and bear the edges of the frame (Figure 1c). Samples were dried for 20 min at 60 °C in a dehydrator (Figure 1d). The tape as well as the central substrate was carefully removed (Figure 1e) to leave the film adhered on both edges and suspended in the core (Figure 1f).

The CNC–MXene dispersion was prepared as described previously (Voignac et al., 2024).^36^ CNC powder (Celluforce, Canada) was dispersed in a 2 wt % solution using probe sonication (QSonica 500) for 10 min at 60% amplitude on a 1 s on, 1 s off regime. A 1.79 wt % MXene colloidal solution, prepared from a Ti_3_AlC_2_ MAX phase as described in Voignac et al., 2024, was added to the CNC dispersion in a 1:3 MXene:CNC ratio.^36^ The solution was then sonicated in a bath sonicator to prevent damage to the MXene flakes and centrifuged to remove any air bubbles. Characterization of the synthesized MXene is provided in the Supporting Information (SI). An intended homemade system was constructed to control the current and measure the voltage drop across the device under various radial acceleration profiles. The custom measurement system was engineered and designed consisting of several key components, shown in Figure 2a: (I) Rotating arm driven by a brushless direct current (BLDC) motor coupled to a 1:10 gearbox via a shaft. The device being tested is then secured to the rotating arm at a predetermined position relative to the center of rotation and subjected to the centrifugal force generated by the rotation. (II) XYZ stage, located on the rotating arm, for positioning the device in different orientations relative to the axis of rotation. (III) MEMS reference accelerometer, located at the same location as the tested device on the rotating arm, to measure independently and compare the radial acceleration. (IV) Slip ring, an electric-mechanical component that allows the transmission of electrical signals and power between the stationary and the rotating part of the system. In this case, it acts as a rotary electrical interface between the device and the stationary source meter unit, allowing the measurement of the voltage drop across the device while it rotates. (V) The measurements are done by applying a constant current and measuring the voltage drop across the CMHF.

A closer look at the experimental scheme is shown in Figure 2b-i. In the absence of an external force, a constant current is applied to the suspended CMHF through two of the leads, while the voltage drop is continuously monitored across a separate pair of leads in a four-probe configuration. This setup enables a precise voltage measurement by minimizing contact resistance. In Figure 2b-ii it is shown that when mechanical force is applied such as during acceleration, the resulting deformation of the film induces a piezoelectric field, which gates the charge transport pathways and modulates the local conductivity, leading to a measurable change in the voltage signal. This modulation originates from a nonlinear gating effect, analogous to the operation of a field-effect transistor. In the rotational setup, the applied acceleration is radial and directed toward the center of rotation, while the corresponding centrifugal force acts outward. The magnitude of the acceleration experienced by the CMHF is directly proportional to the tangential speed of the rotating arm, allowing controlled modulation of the mechanical stimulus.

Initially, the device was oriented in such a way that the force will pull the CMHF out of the device plane (defined as the +*z* direction, see Figure 4b), while a constant current of 1, 10, or 20 mA was applied to the device. The voltage drop across the CMHF was measured as a function of time with a time resolution of 10 ms while a radial acceleration profile was applied by controlling the speed of the rotating arm. The acceleration profile consists of five phases: a 10 s idle time, a 2 s rotation frequency acceleration, an 8 s constant rotation frequency, a 2 s rotation frequency ramp down time, and another 10 s of idle time (see SI Figure S2). During the rotation frequency stage, the arm rotates at a constant desired speed, resulting in a known radial acceleration and centrifugal force being applied to the tested acceleration sensor. Figures 3a, 3b, and 3c show typical responses of the CMHF device when subjected to a 10*g* radial acceleration profile, at an applied constant current of 1 mA, 10 mA, or 20 mA, respectively. Additionally, the radial acceleration was measured in parallel using a commercially available MEMS accelerometer, for reference. Due to the capacitive nature of the CMHF device, applying a constant current causes continuous charging, leading to a gradual decrease in the measured voltage drop over time, which appears to follow a linear trend (see SI Figure S3a). To better interpret the results during postprocessing, a linear fit is applied to the data, and the corresponding slope is subtracted. This approach effectively “straightens” the resulting curve, making the voltage response of the device more intuitive and easier to analyze (see SI Figure S3b). During the first stage (idle time), where the arm is at rest, the voltage across the device remains constant as the CMHF remains still. During acceleration at the second stage (rise time), the voltage across the device undergoes a rapid decline. The voltage stops declining at the phase where constant rotation frequency is applied; thus constant centrifugal force is acting on the CMHF, bending and applying stress to the CMHF. In the fall time and final stage (idle time), when the arm comes to a stop, the voltage across the device returns to a constant value. In that time, no rotational forces are acting on the CMHF and the film is stable, as reflected in the constant voltage. To determine the optimal working current for the CMHF device, signal-to-noise ratio (SNR) analysis was performed. This was done by defining the inherent device noise as half of the peak-to-peak amplitude of the voltage measured during the idle time and the signal amplitude as the peak-to-peak amplitude of the voltage measured during the constant rotation frequency stage. The SNR was calculated for the different applied constant currents (Figure 3d). From the measured currents, the optimal working current is 20 mA, as it results in a higher SNR. Higher currents were also measured, and while the SNR improved with increased applied current, the CMHF stability was lower as the devices were eventually shorted out after a few cycles. This failure is likely due to Joule heating, which causes the CNC to degrade and burn, ultimately disrupting the electrical connection and preventing further signal acquisition.

**Figure 3:**
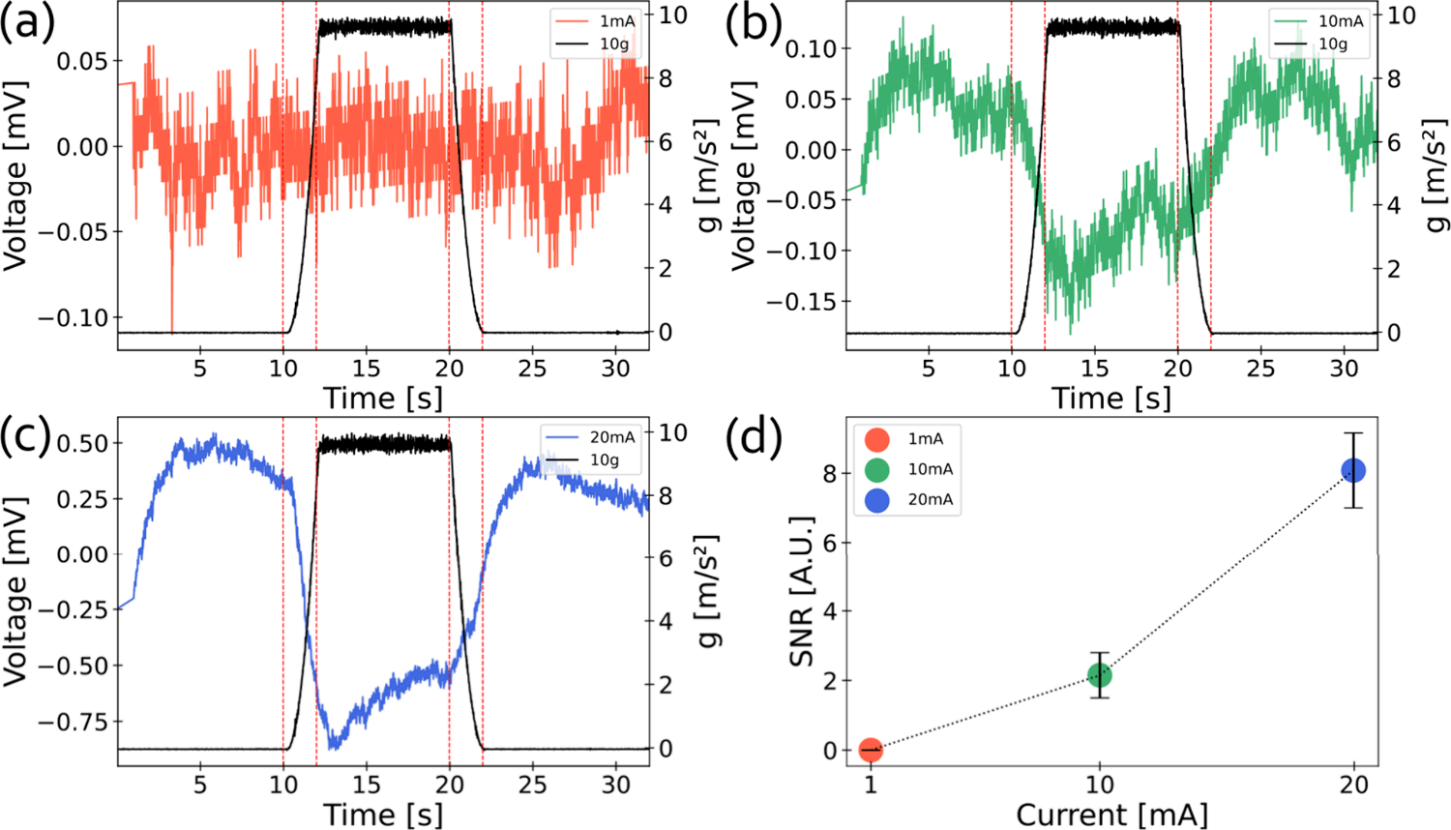
Typical CNC–MXene hybrid film device response to a radial acceleration profile measured by a MEMS accelerometer in black, representing voltage vs time, *V*(*t*), and acceleration vs time, *a*(*t*), at an applied current of (a) 1 mA and 10*g*, (b) 10 mA and 10*g*, and (c) 20 mA and 10*g*, respectively. Red vertical lines correspond to the transition points of the acceleration profile. (d) Signal to noise ratio (SNR) vs current at 10*g*.

**Figure 4:**
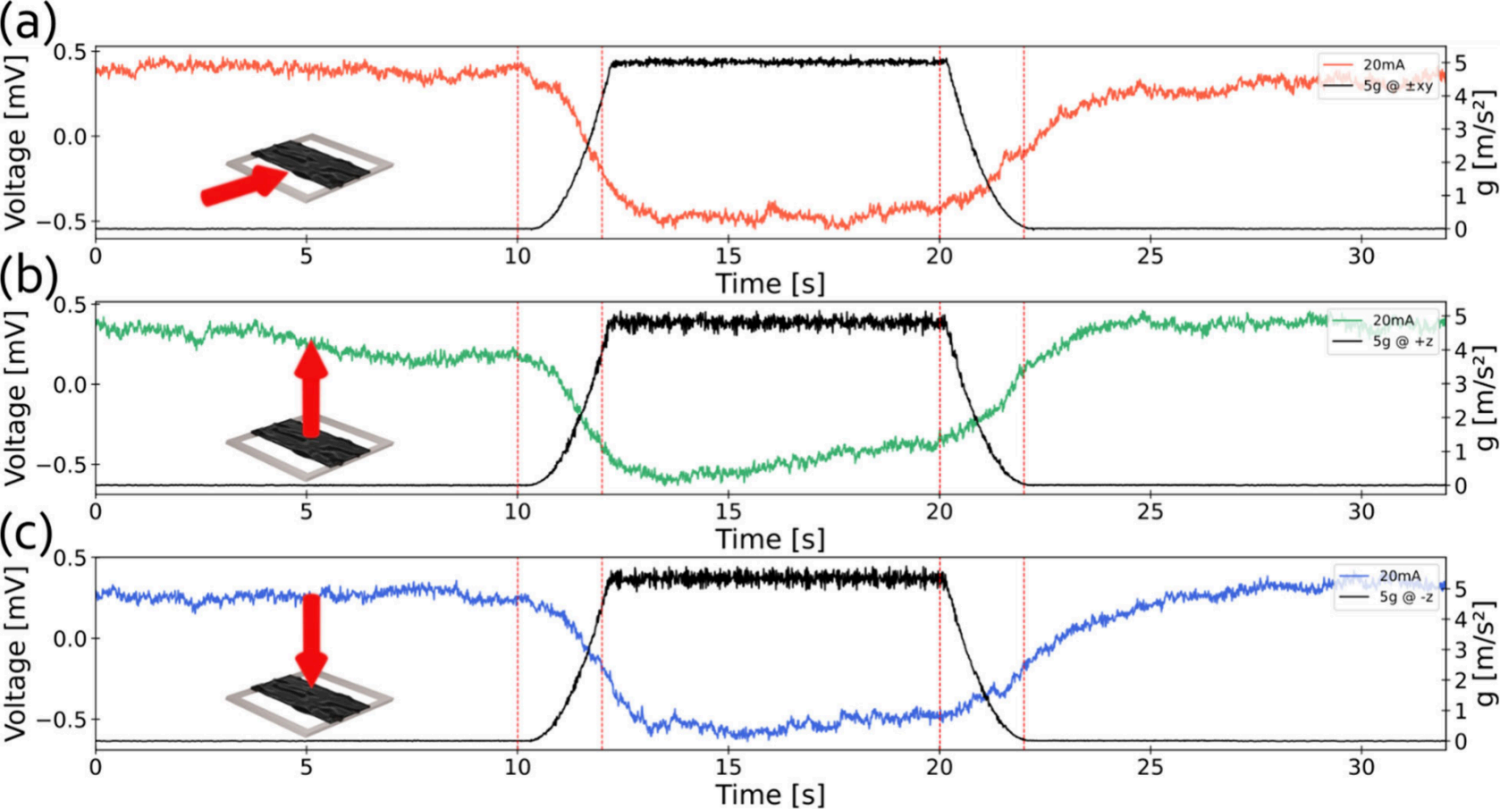
CNC–MXene hybrid film device voltage vs time response to radial acceleration in various placement orientations. *V*(*t*) and *a*(*t*) under 20 mA at (a) 5*g*, ±*xy* orientation, (b) 5*g*, +*z* orientation, and (c) 5*g*, −*z* orientation. Red vertical lines correspond to the transition points of the acceleration profile.

The device response to radial acceleration in all possible rotational axes was characterized for ±*z* and ±*xy* orientations as depicted in Figure 4. The same measurement protocol as that described previously was repeated for each orientation. CMHF device voltage response with time to radial acceleration in various orientations was obtained at 5*g* acceleration profile and at various orientations (±*z* and ±*xy*), for a 20 mA applied constant current (Figures 4a–c). In addition, the radial acceleration as a function of time was measured with a commercially available MEMS accelerometer for reference. For all tested orientations, the device exhibits a consistent response for the applied radial acceleration profiles tested, with voltage decreasing during the acceleration stage and stabilizing during the constant force stage. This unique “apathic to direction” response is worth further future studies, and we hypothesize that the comparable response observed across all orientations arises from the symmetric geometry of the CMHF device, combined with the intermittently distributed conductive MXene layers that facilitate uniform charge transport throughout the film. This structural and electrical symmetry likely yields a strain distribution and corresponding resistance modulation that remain largely invariant with respect to the direction and point of applied force. Furthermore, the mechanical response of the suspended CNC–MXene composite may be effectively isotropic, potentially due to the random orientation of the MXene flakes and uniform boundary constraints, leading to a similar deformation behavior under multidirectional loading. To enhance directional sensitivity, we plan to introduce an asymmetric design combined with different MXene:CNC ratios in future work, which we anticipate will enable differentiation between the axes along which the force is applied, allowing for precise directional acceleration sensing. Regarding response time, we note that our device operates on the basis of the piezoelectric effect in the CNC–MXene hybrid film, which is inherently fast and suitable for capturing transient signals. From our current measurements, the device shows an immediate voltage response upon acceleration, without an observable delay within the resolution of our setup. Specifically, the measurements were performed with a time resolution of 0.01 s, which represents the fastest sampling rate allowed by our present setup. Within this time scale, the device appears to react instantaneously to the applied acceleration. While a full frequency-domain characterization is part of our planned future studies, the present results indicate that the device is not restricted to quasi-static or low-frequency motion. To further investigate and characterize the CMHF device, the same measurement procedure described above was repeated multiple times and continuously. The typical voltage response vs time of the CMHF device to radial acceleration in the +*z* orientation at 1*g*, 5*g*, and 10*g* was recorded and is presented in Figures 5a–c, respectively. The relationship between voltage response amplitude and acceleration magnitude is illustrated in Figure 5d, showing a clear trend that higher acceleration levels result in an increased voltage response amplitude, as expected. The CMHF exhibits a sensitivity of 0.053 mV/g, determined by the average of two data sets from devices A and B. The detection limit of the device is estimated to be ∼0.5*g*, since at 0.1*g* the signal is dominated by noise, whereas from 0.5*g* and above the response becomes stable and follows a linear trend.

**Figure 5:**
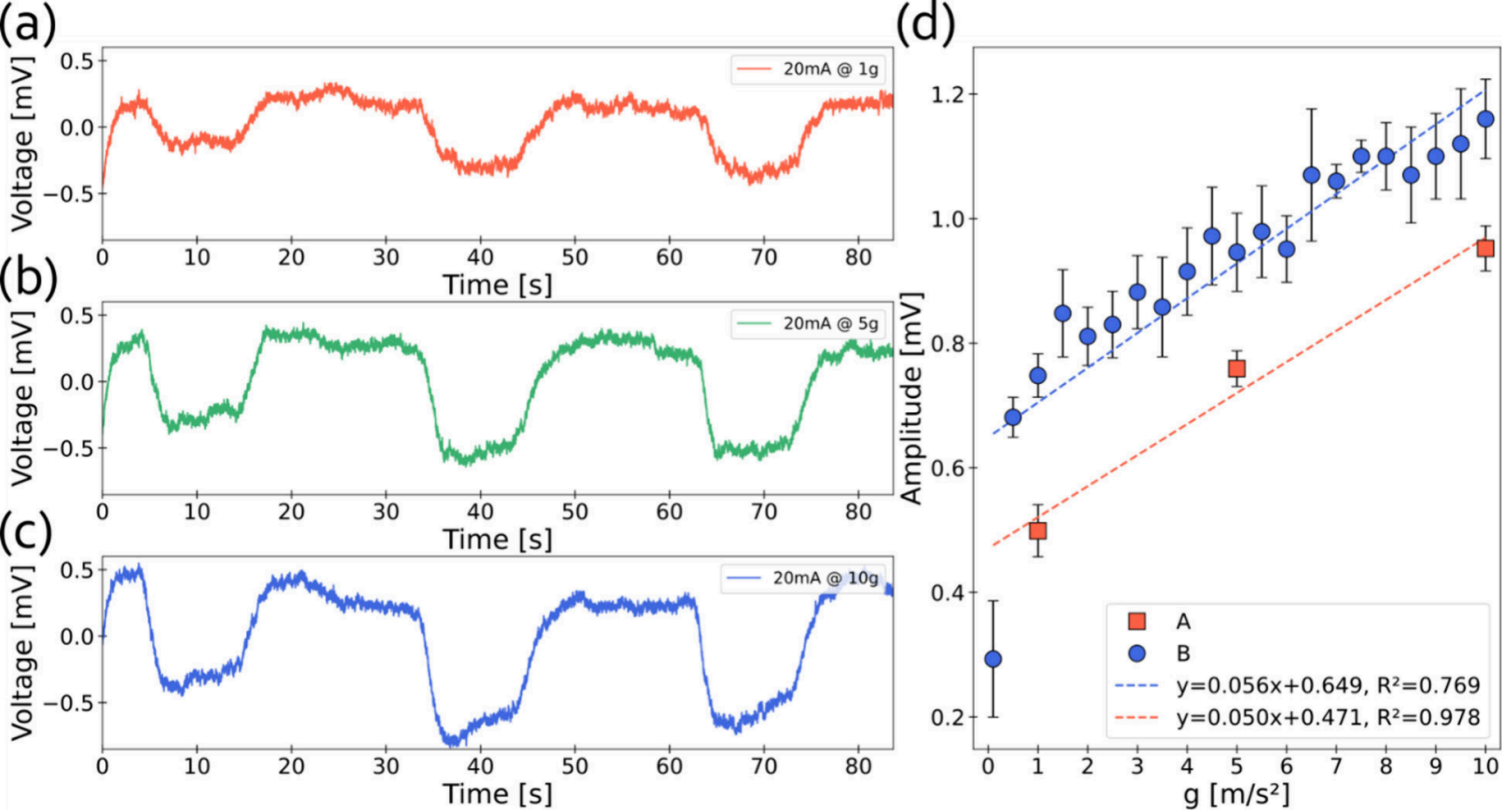
Typical voltage response to various radial acceleration magnitudes at the +*z* orientation. *V*(*t*) at 20 mA. (a) 1*g*. (b) 5*g*. (c) 10*g*. (d) Voltage response amplitude to radial acceleration. Error bars represent the standard deviations of the average voltage response amplitude.

Table 1 summarizes the key performance metrics of the CMHF sensor in comparison with representative flexible piezoelectric sensors and established commercial devices. While the sensitivity of the CMHF device (0.053 mV/g) is lower than that of polymer-based PVDF sensors and commercial piezo-ceramic sensors, the results clearly demonstrate that the CMHF can consistently transduce acceleration into measurable electrical signals. The device was characterized for up to 10*g*, which corresponds to the upper limit of our custom-built measurement setup; however, the stable signal response obtained at this range strongly suggests that the CMHF sensor is capable of operating at higher accelerations. Importantly, the CMHF device achieves this performance with an operating range of 0.5–10*g* and an ultralow power consumption of ∼10 μW, which is orders of magnitude lower than conventional MEMS (∼0.7 mW) and piezo-ceramic (∼60 mW) accelerometers. Moreover, unlike conventional piezoelectric accelerometers that rely on direct charge readout, the CMHF sensor exploits the piezoelectric response of CNC films as a gating voltage on the conductive MXene network. This mechanism allows acceleration sensing, even though CNC is not considered a high-performance piezoelectric material. While the presented results do not rely solely on the piezoelectric properties of the complex but a piezo-induced gating effect, others have reported piezoelectric coefficients of *d*_33_ = 19.3 ± 2.9 pm/V for vertically aligned CNC films^40^ and *d*_25_ = 2.1 Å/V for shear piezoelectric constant.^41^ Finally, the device is fabricated from biodegradable, sustainable, and flexible materials, distinguishing it from both rigid MEMS and inorganic piezo-ceramic sensors. CNC is known for its low thermal expansion coefficient, suggesting minimal impact from environmental temperature changes.^42^ This could be a path for future work on *in situ* devices under extreme conditions. The hygroscopic nature of cellulose may influence results but can also be mediated through vacuum packaging or cross-linking methods on the free surface hydroxyl groups such as carboxylic acid cross-linking.^43^ Taken together, these features position the CMHF sensor as a proof-of-concept platform that although not yet optimized for maximum sensitivity, opens a new direction for low-power, low-cost, and environmentally friendly acceleration sensing technologies.

**Table 1:** Performance Comparison to Other Accelerometers

Device (Year)	Material	Sensitivity	Operating Range	Power Consumption
CMHF (this work)	CNC–MXene hybrid (flexible)	0.053 mV/g	0.5–10*g* ^a^	10 μW
Wu et al., Sens. Actuators A (2025)^37^	PVDF + ZnO composite (flexible)	4.558 V/g	N/A	Passive
Ge & Cretu, Microsystems & Nanoeng. (2024)^38^	PVDF (polymer, flexible)	29.45 pC/g	±10*g*	Passive
Ge & Cretu, Microsystems & Nanoeng. (2023)^39^	PVDF (polymer, flexible)	21.82 pC/g (≈126.3 mV/g)	±6*g*	Passive
PCB Piezotronics 352C33 (datasheet)	Piezo-ceramic (shear, PZT)	100 mV/g	±50*g*	60 mW
Analog Devices ADXL355 (datasheet)	Silicon MEMS (capacitive)	Digital	±2/4/8*g*	0.7 mW

a The device was characterized up to 10*g*.

In summary, a self-assembled acceleration sensor based on a CNC–MXene hybrid film (CMHF) is presented, leveraging the unique piezoelectric and conductive properties of its nanoscale components. This novel device is straightforward to fabricate, combining standard top-down methods for macroscale components with bottom-up self-assembly for the active sensing film. The sensor demonstrates sensitivity to acceleration across multiple axes and a unique “apathic to direction” response, offering a competitive alternative to conventional MEMS accelerometers. We believe that such a device has potential applications in a wide range of fields, including aerospace, automotive safety, structural health monitoring, and biomedical engineering. Its ability to detect accelerations with high precision makes it particularly suitable for vibration analysis in mechanical systems, motion tracking in robotics, and monitoring dynamic forces in wearable technology. Additionally, its low operating current and voltage contribute to reduced power consumption (10 MW), making it ideal for energy-efficient applications, such as implantable medical devices and remote sensing systems. Furthermore, the biodegradable nature of CNC makes the sensor an environmentally friendly alternative, which is particularly beneficial for transient electronics, disposable biomedical sensors, and sustainable monitoring systems. Beyond acceleration sensing, the CMHF device also shows promise for soft robotics applications, demonstrating sensitivity to both subtle movements and very light contact. Overall, this new CNC–MXene-based acceleration sensor presents a promising avenue for next-generation motion sensing technologies.

## Supplementary Material



## Notes

Characterization of synthesized MXene, rotation speed calculation, acceleration profile, detrended voltage curve, repeated mechanical stress data, *I*–*V* curve, and additional results (PDF)

## References

[1] Mohankumar P., Ajayan J., Yasodharan R., Devendran P., Sambasivam R. (2019). “A review of micromachined sensors for automotive
applications,”. Measurement.

[2] Troiano R. P., McClain J. J., Brychta R. J., Chen K. Y. (2014). “Evolution
of accelerometer methods for physical activity research,”. Br J. Sports Med..

[3] Ge C., Cretu E. (2023). “A polymeric
piezoelectric MEMS accelerometer with high sensitivity,
low noise density, and an innovative manufacturing approach,”. Microsystems & Nanoengineering 2023 9:1.

[4] Hindrichsen C. C., Almind N. S., Brodersen S. H., Lou-Møller R., Hansen K., Thomsen E. V. (2010). “Triaxial
MEMS accelerometer
with screen printed PZT thick film,”. J. Electroceram.

[5] Mo J., Shankar S., Pezone R., Zhang G., Vollebregt S. (2024). “A
high aspect ratio surface micromachined accelerometer based on a SiC-CNT
composite material,”. Microsystems &
Nanoengineering 2024 10:1.

[6] Algamili A. S. (2021). “A Review of
Actuation and Sensing Mechanisms in MEMS-Based
Sensor Devices,”. Nanoscale Res. Lett..

[7] D’Alessandro A., Scudero S., Vitale G. (2019). “A Review of the Capacitive
MEMS for Seismology,”. Sensors 2019,
Vol. 19, Page 3093.

[8] Fraga M. A., Furlan H., Pessoa R. S., Massi M. (2014). “Wide bandgap
semiconductor thin films for piezoelectric and piezoresistive MEMS
sensors applied at high temperatures: An overview,”. Microsystem Technologies.

[9] Herrera-May, A. L. , Aguilera-Cortés, L. A. , García-Ramírez, P. J. , Manjarrez, E. , “Resonant magnetic field sensors based on MEMS technology,” Sensors 2009, 9, 7785–7813 10.3390/s91007785. 22408480 PMC3292083

[10] Ramakrishnan, J. ; Gaurav, P. T. R. ; Chandar, N. S. ; Sudharsan, N. M. , “Structural design, analysis and DOE of MEMS-based capacitive accelerometer for automotive airbag application,” Microsystem Technologies 27, 3, 2021, 763 10.1007/s00542-020-04979-3.

[11] Li R. J., Lei Y. J., Chang Z. X., Zhang L. S., Fan K. C. (2018). “Development
of a High-Sensitivity Optical Accelerometer for Low-Frequency Vibration
Measurement,”. Sensors 2018, Vol. 18,
Page 2910.

[12] El-Diasty M., Pagiatakis S. (2009). “A
Rigorous Temperature-Dependent Stochastic
Modelling and Testing for MEMS-Based Inertial Sensor Errors,”. Sensors.

[13] Han S., Meng Z., Omisore O., Akinyemi T., Yan Y. (2020). “Random
Error Reduction Algorithms for MEMS Inertial Sensor Accuracy Improvement—A
Review,”. Micromachines 2020, Vol. 11,
Page 1021.

[14] Yoon Y. K., Park J. H., Allen M. G. (2006). “Multidirectional
UV lithography
for complex 3-D MEMS structures,”. Journal
of Microelectromechanical Systems.

[15] Liao Y. (2023). “A Review of
Flexible Acceleration Sensors Based on Piezoelectric
Materials: Performance Characterization, Parametric Analysis, Frontier
Technologies, and Applications,”. Coatings
2023, Vol. 13, Page 1252.

[16] Xu J. (2022). “High-Frequency Vibration Analysis of Piezoelectric
Array
Sensor under Lateral-Field-Excitation Based on Crystals with 3 m Point
Group,”. Sensors 2022, Vol. 22, Page
3596.

[17] Gong X., Kuo Y. C., Zhou G., Wu W. J., Liao W. H. (2023). “An
aerosol deposition based MEMS piezoelectric accelerometer for low
noise measurement,”. Microsystems &
Nanoengineering 2023 9:1.

[18] Ogbonna V. E., Popoola A. P. I., Popoola O. M. (2022). “Piezoelectric
ceramic materials
on transducer technology for energy harvesting: A review,”. Front Energy Res..

[19] Wang G. (2018). “Flexible pressure sensor based on PVDF nanofiber,”. Sens Actuators A Phys..

[20] Ali G., Mohd-Yasin F. (2024). “Comprehensive Noise Modeling of Piezoelectric
Charge Accelerometer with Signal Conditioning Circuit,”. Micromachines 2024, Vol. 15, Page 283.

[21] Luo, Q. ; Shen, H. ; Zhou, G. ; Xu, X. “Dielectric Properties of Cellulose and Nanocellulose-Based Materials,” Carbohydrate Polymers 2023, 303, 120449 10.1016/j.carbpol.2022.120449. 36657840

[22] Abitbol, T. ; , “Nanocellulose, a tiny fiber with huge applications,” Curr. Opin. Biotechnol. 2016, 39, 76 10.1016/j.copbio.2016.01.002. 26930621

[23] Kose, O. ; Tran, A. ; Lewis, L. ; Hamad, W. Y. ; MacLachlan, M. J. “Unwinding a spiral of cellulose nanocrystals for stimuli-responsive stretchable optics,” Nat. Commun. 10, 1, 2019, 10.1038/s41467-019-08351-6. PMC6355765 30705267

[24] Li F., Biagioni P., Bollani M., Maccagnan A., Piergiovanni L. (2013). “Multi-functional coating
of cellulose nanocrystals
for flexible packaging applications,”. Cellulose.

[25] Zhao, D. ; Zhu, Y. ; Cheng, W. ; Chen, W. ; Wu, Y. ; Yu, H. “Cellulose-Based Flexible Functional Materials for Emerging Intelligent Electronics,” Adv. Mater., 33, 28, 2021, 10.1002/adma.202000619. 32310313

[26] Pérez J., Muñoz-Dorado J., De La Rubia T., Martínez J. (2002). “Biodegradation
and biological treatments of cellulose, hemicellulose and lignin:
An overview,”. Sociedad Espanola de Microbiologia.

[27] Petritz A. (2013). “Cellulose as
biodegradable high-k dielectric layer in organic
complementary inverters,”. Appl. Phys.
Lett..

[28] Tran A., Boott C. E., MacLachlan M. J. (2020). “Understanding
the Self-Assembly
of Cellulose Nanocrystals—Toward Chiral Photonic Materials,”. Adv. Mater..

[29] Seo S. M., Lee J. W., Shin J., Tak J. H., Hyun J., Park I. K. (2021). “Development of cellulose nanocrystal-stabilized
Pickering emulsions of massoia and nutmeg essential oils for the control
of Aedes albopictus,”. Scientific Reports
2021 11:1.

[30] Yu B. (2019). “Fabrication of PLA/CNC/CNT conductive composites
for high
electromagnetic interference shielding based on Pickering emulsions
method,”. Compos Part A Appl. Sci. Manuf.

[31] Csoka L., Hoeger I. C., Rojas O. J., Peszlen I., Pawlak J. J., Peralta P. N. (2012). “Piezoelectric
effect of cellulose nanocrystals
thin films,”. ACS Macro Lett..

[32] Voignac, D. ; Belsey, S. ; Wermter, E. ; Paltiel, Y. ; Shoseyov, O. “Biobased Electronics: Tunable Dielectric and Piezoelectric Cellulose Nanocrystal—Protein Films,” Nanomaterials 13, 15, 2023, 2258 10.3390/nano13152258.37570575 PMC10421335

[33] Al-Bustami H. (2022). “Spin-Induced
Organization of Cellulose Nanocrystals,”. Biomacromolecules.

[34] Anasori B., Lukatskaya M. R., Gogotsi Y. (2017). “2D metal carbides and nitrides
(MXenes) for energy storage,”. Nature
Reviews Materials 2017 2:2.

[35] Iqbal A., Hong J., Ko T. Y., Koo C. M. (2021). “Improving
oxidation stability of 2D MXenes: synthesis, storage media, and conditions,”. Nano Converg.

[36] Voignac D. (2024). “MXene-CNC super performing composite films
for flexible and
degradable electronics,”. Carbon N Y.

[37] Wu B. (2025). “Ultra-sensitive
flexible accelerometer based on PVDF for
self-powered wireless sensing system,”. Sens Actuators A Phys..

[38] Ge C., Cretu E. (2024). “Polymeric
piezoelectric accelerometers with high sensitivity,
broad bandwidth, and low noise density for organic electronics and
wearable microsystems,”. Microsyst Nanoeng.

[39] Ge C., Cretu E. (2023). “A
polymeric piezoelectric MEMS accelerometer with high sensitivity,
low noise density, and an innovative manufacturing approach,”. Microsyst Nanoeng.

[40] Wang J. (2020). “Piezoelectric
Nanocellulose Thin Film with Large-Scale Vertical
Crystal Alignment,”. ACS Appl. Mater.
Interfaces.

[41] Csoka L., Hoeger I. C., Rojas O. J., Peszlen I., Pawlak J. J., Peralta P. N. (2012). “Piezoelectric effect of cellulose nanocrystals
thin films,”. ACS Macro Lett..

[42] Diaz J. A., Wu X., Martini A., Youngblood J. P., Moon R. J. (2013). “Thermal
expansion of self-organized and shear-oriented cellulose nanocrystal
films,”. Biomacromolecules.

[43] Ben
Shalom T., Belsey S., Chasnitsky M., Shoseyov O. (2021). “Cellulose nanocrystals and corn zein oxygen
and water vapor barrier biocomposite films,”. Nanomaterials.

