# Supplementary material for: Self-Assembled Cellulose Nanocrystal–MXene Hybrid Film for Acceleration Sensing
Source: Nano Lett. 2025 Oct 29;25(45):16068–75. doi: 10.1021/acs.nanolett.5c02824 (PMC12616782; doi:10.1021/acs.nanolett.5c02824)
Supplement: Supplementary file 1 [file nl5c02824_si_001.pdf]

Self-Assembled Cellulose Nanocrystal-MXene Hybrid Film for Acceleration Sensing

Omer Shoseyov[a,c], Daniel Voignac[b,c], Shylee Belsey[b], Danielle Sviri[d], Shira Yochelis[a,c], Maxim Sokol[d], Oded Shoseyov[b,c,*] and Yossi Paltiel[a,c,*]

a) Institute of Applied Physics, The Hebrew University of Jerusalem, Jerusalem 9190401, Israel.
b) The Robert H. Smith Institute of Plant Sciences and Genetics in Agriculture, Robert H. Smith Faculty of Agriculture, Food and Environment, The Hebrew University of Jerusalem, Rehovot 7612001, Israel.
c) The Center for Nanoscience and Nanotechnology, The Hebrew University of Jerusalem, Jerusalem 9190401, Israel.
d) Department of Materials Science and Engineering, Tel Aviv University, Ramat Aviv 6997801, Israel.

Supporting Information

A) Characterization of the synthesized MXene

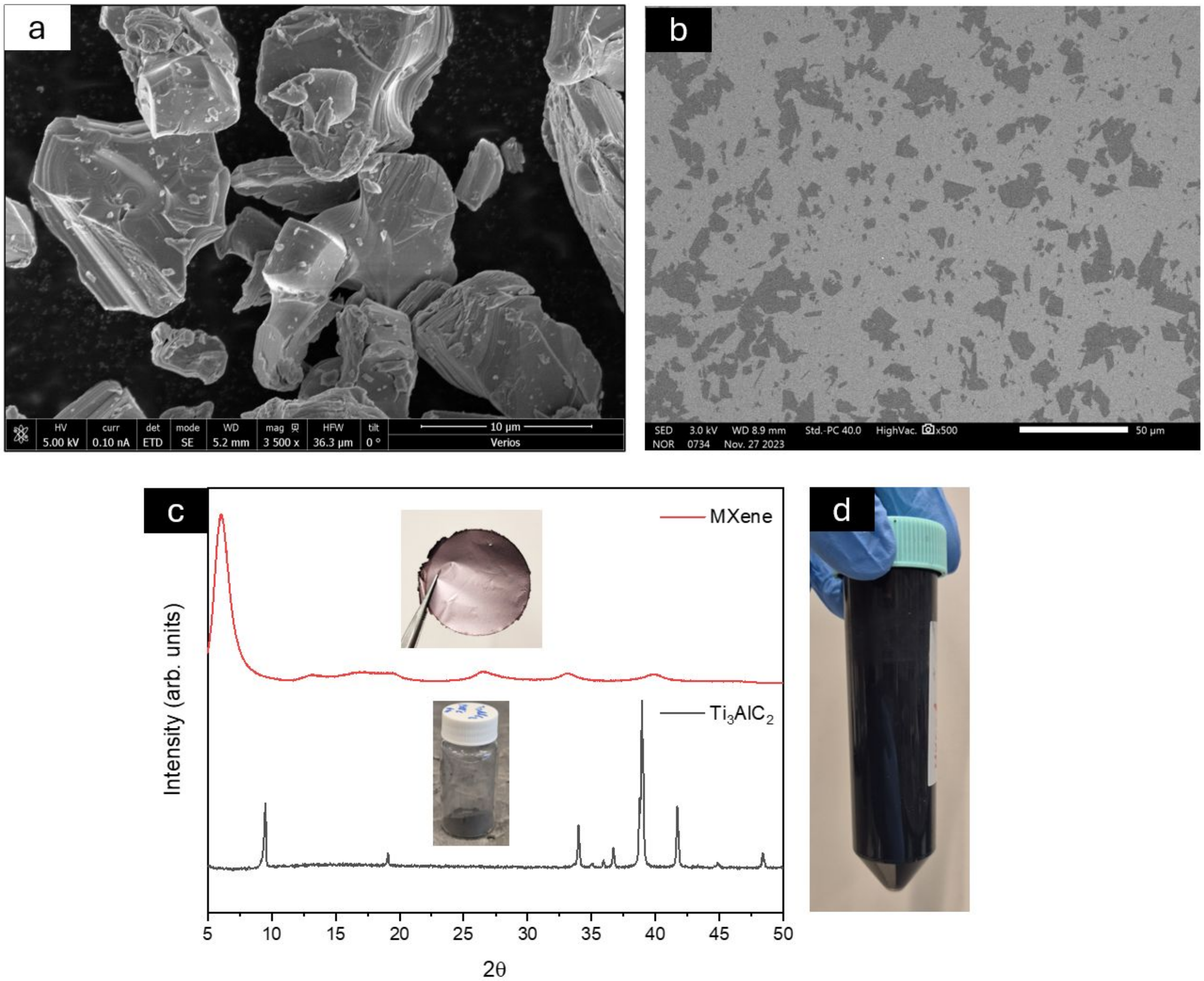

Figure S1. (*a) SEM image of Ti_3AlC_2 MAX phase particles, (b) SEM image of single-layer MXene flakes deposited on a silicon wafer, (c) XRD patterns of Ti_3AlC_2 MAX phase (black) and delaminated MXene (red), with insets showing a free-standing MXene film (top) and vial containing MAX powder (bottom), (d) Photograph of a centrifuge tube containing a MXene colloidal solution after exfoliation.*

B) Rotation Speed Calculation

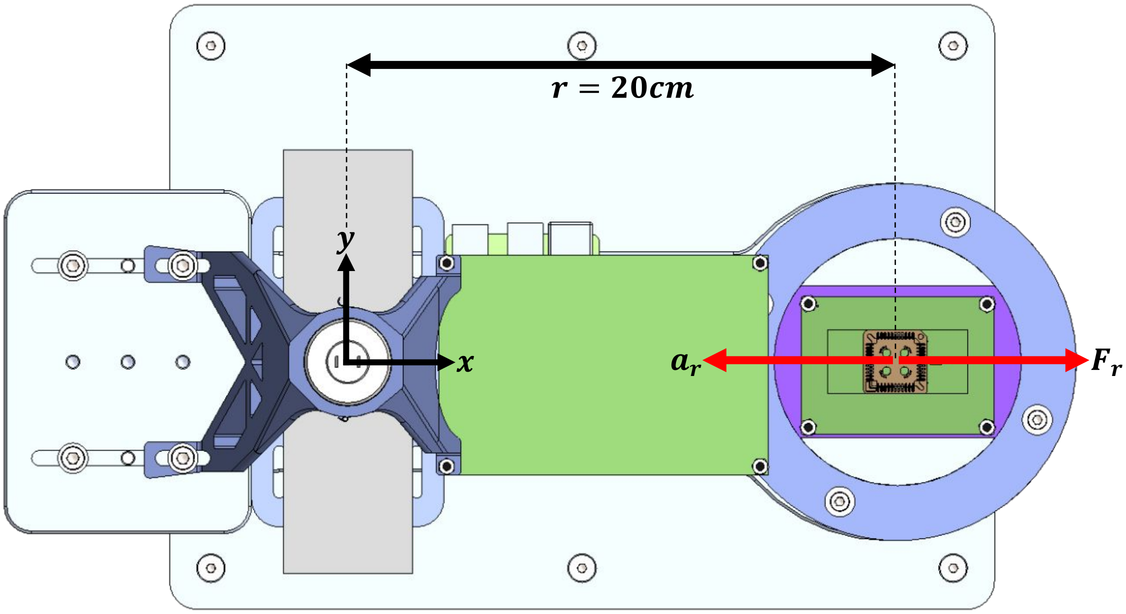

Figure S2. *Top view of the measurement system.*

Figure S2 depicts the position of the device relative to the center of rotation denoted by r, where the device experiences a radial acceleration towards the center of rotation due to the rotation of the arm. This radial acceleration results in a centrifugal force acting in the opposite direction, and by controlling the speed of the rotating arm, the magnitude of both, radial acceleration and centrifugal force can be controlled.

The calculation of the rotation speed is as follows:

$$a_r = r\omega^2 \left[\frac{m}{s^2}\right], \omega = \frac{v}{r}\left[\frac{rad}{s}\right], g = 9.81 \left[\frac{m}{s^2}\right]$$

$$\omega_{ng} = \sqrt{\frac{a_r}{r}} = \sqrt{\frac{n \cdot g}{r}} \cdot \frac{60}{2\pi}[RPM], n = 0, 1, 2 \ldots$$

A few examples are shown in the following table:

$a_r\left[\frac{m}{s^2}\right]$	ω_{ng} $[RPM]$	f_{ng} $[Hz]$
$0.1g$	21.14	0.352
$0.5g$	47.29	0.788
$1g$	66.87	1.114
$3g$	115.83	1.930
$5g$	149.54	2.492
$10g$	211.49	3.524

C) Acceleration Profile

In Figure S3, a(t), the acceleration profile as a function of time is shown.

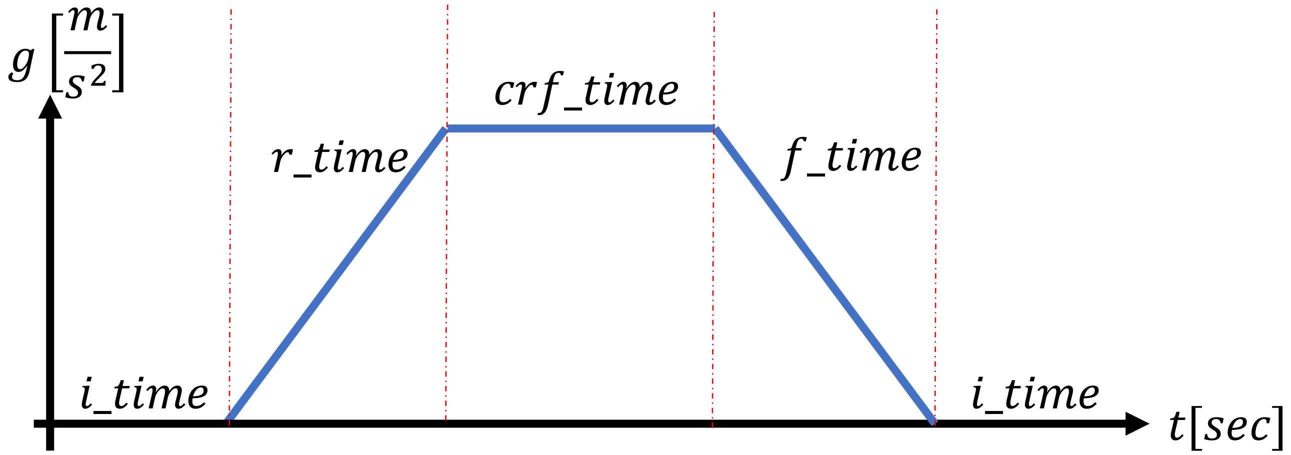

Figure S3. *Acceleration profile. i_time: the arm is at rest. r_time: the arm accelerates to the final rotation frequency. crf_time: the arm rotates at a constant final frequency. f_time: the arm deaccelerates to a stop.*

D) Detrended Voltage Curve

In Figure S4, V(t), before and after the slope has been removed is shown.

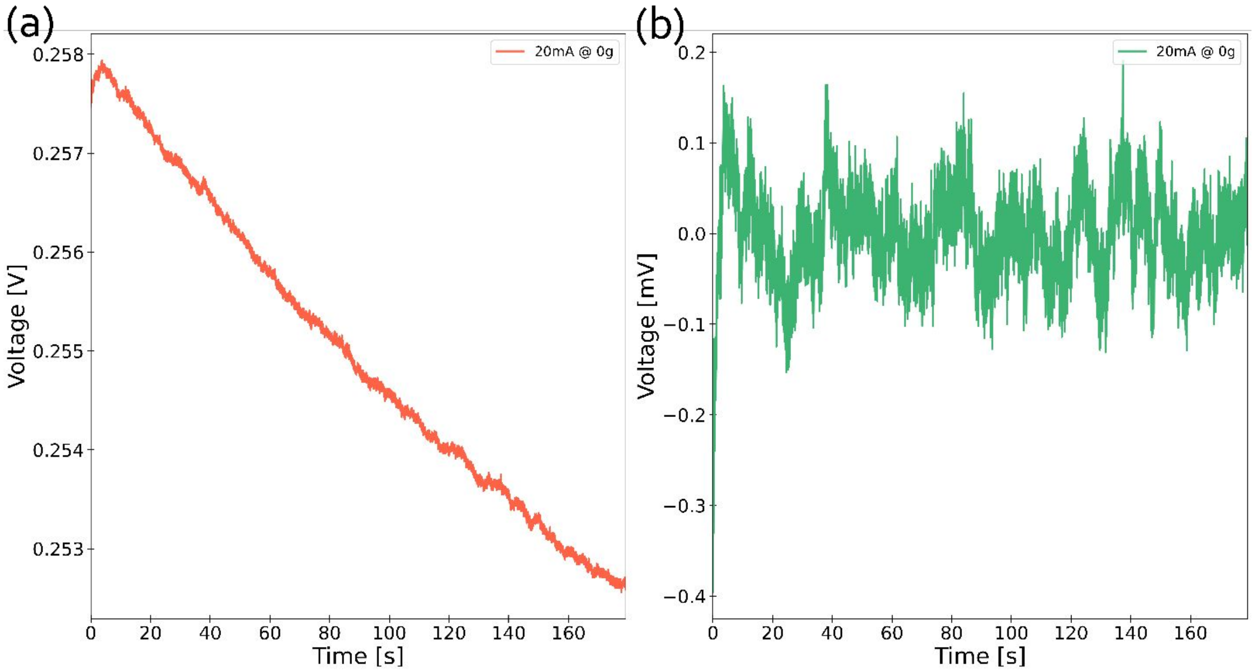

Figure S4. *Voltage response with no acceleration. (a) Original data. (b) Detrended data.*

Linear fit is applied to the data, and the corresponding slope is subtracted. This approach effectively "straightens" the resulting curve, making the voltage response of the device more intuitive and easier to analyze.

E) Repeated Mechanical Stress

In Figure S5, V(t), is shown. The device was subjected it to 100 repeated acceleration-deceleration cycles. The sensor maintained consistent performance throughout these cycles, indicating robust stability under repeated mechanical stress. The main observed change over time is in the slope of the measured voltage response: at short timescales the slope decreases linearly, while over longer times it follows an exponential decay, consistent with the expected behaviour of a capacitive system.

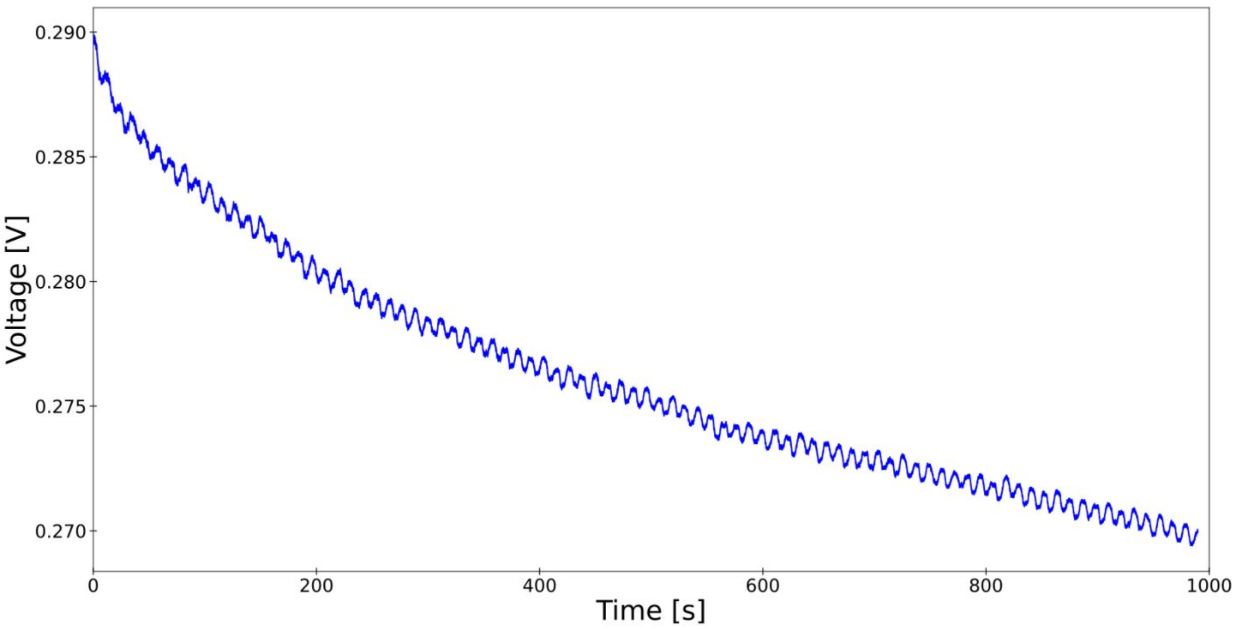

Figure S5. *Voltage response to repeated mechanical stress.*

F) I-V Curve

Figure S6 depicts the Current-voltage characteristics of the CMHF film.

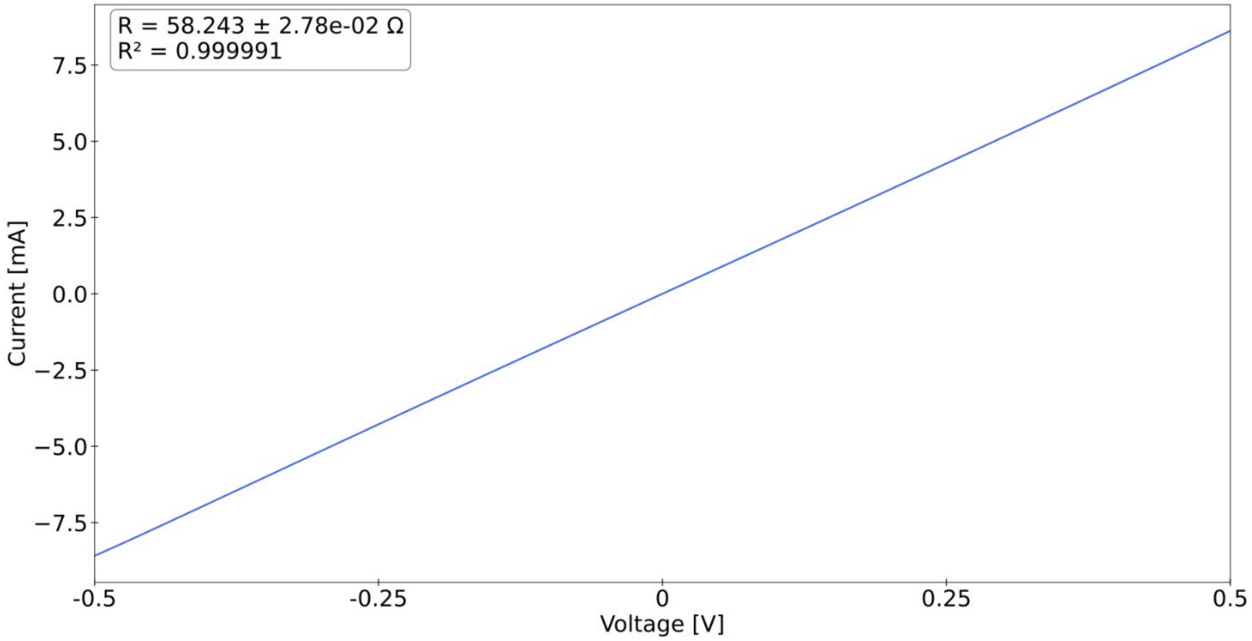

Figure S6. *Current-voltage characteristics of the CMHF film. The plot shows the average current response (blue line) over ten repeated sweeps, with the shaded region representing the standard error of the mean.*

The I-V curve is linear over the tested range of -0.5 V to +0.5 V, indicating ohmic behaviour with negligible contact effects. A linear regression yields a resistance of R ≈ 58.243 Ω ± 2.78e-2 (95% CI), with $R^2 \approx 0.999991$, confirming the reproducibility and stability of the conduction pathway in the hybrid film.

G) Additional Results

Figure S7 depicts three additional devices whose performance aligns with the results reported in the main text.

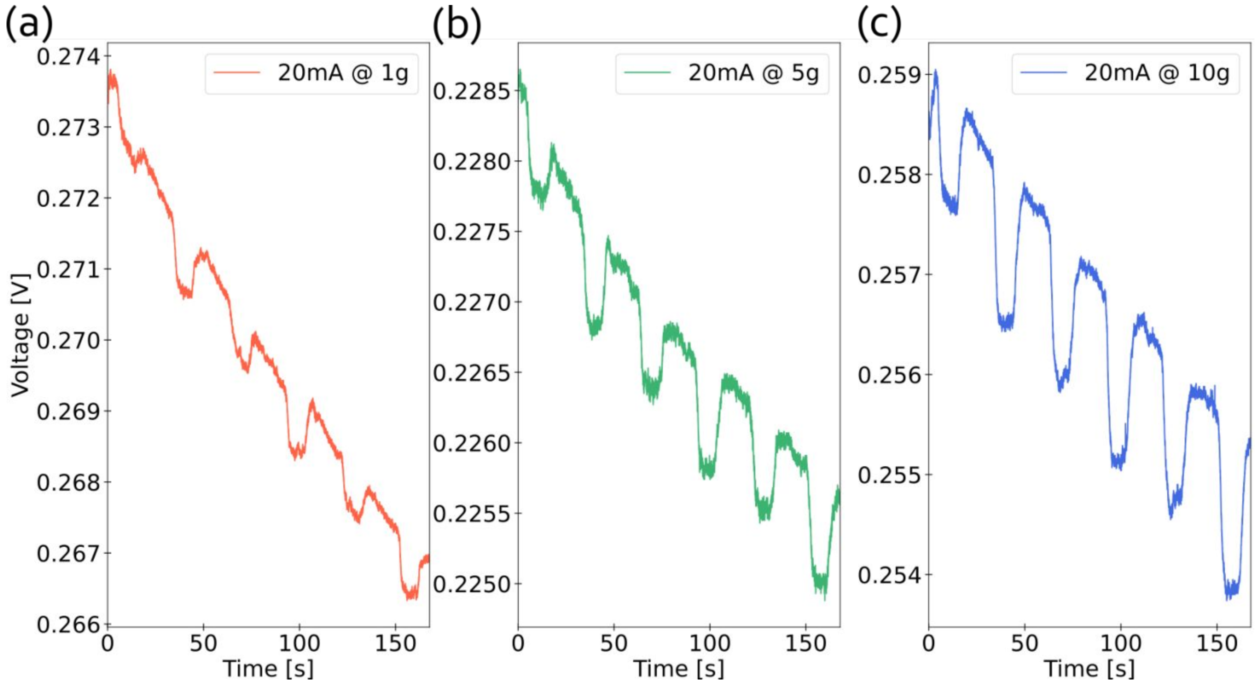

Figure S7. *Typical voltage response to various radial acceleration magnitudes at* $+z$ *orientation. V(t) at 20mA (a) Device C, 1g. (b) Device D, 5g. (c) Device E, 10g.*
